# Metabolomics in Functional Interrogation of Individual Holobiont Members

**DOI:** 10.1128/mSystems.00841-21

**Published:** 2021-08-24

**Authors:** Neha Garg

**Affiliations:** a School of Chemistry and Biochemistry, Georgia Institute of Technology, Atlanta, Georgia, USA; b Center for Microbial Dynamics and Infection, School of Biological Sciences, Georgia Institute of Technology, Atlanta, Georgia, USA; c Petit Institute for Bioengineering and Bioscience, Georgia Institute of Technology, Atlanta, Georgia, USA

**Keywords:** *Symbiodiniaceae*, coral holobiont, dark metabolome, metabolomics, microbiome

## Abstract

Eukaryotes and their environments serve as petri dishes, hosting an abundant and a rich prokaryotic microbiome. The assemblage of a eukaryotic host and its microbiome is referred to as a holobiont. The holobiont’s microbiome interacts within itself, with the environment, and with the host at the chemical level through production of specialized metabolites resulting in homeostasis or dysbiosis. These interactions are triggered by a multitude of factors, such as community composition, age, presence of nutrients, xenobiotics, and change in physical conditions, such as temperature and oxygen. Understanding how holobionts respond and adapt to diverse triggers is necessary to uncover mechanisms of resilience or susceptibility to dysbiosis and to modulate the collective functioning of microbiome in health and disease. This article highlights the challenges associated with uncovering chemical contributions of individual holobiont members and the applicability of metabolomics-based approaches to uncover chemical signatures of microbial processes in the natural environment.

## COMMENTARY

Multipartite interactions within holobiont members are influenced by chemical cross talk between the members of holobiont as well as their interactions with the environment. Additional complexity in these interactions is introduced by the dynamic nature of the members of the holobiont. While some partners are essential and form core symbiotic relationships with the host, the compositions of others vary in response to various environmental and genetic triggers. Thus, the contributions of individual members of the holobiont to host health and the response to environmental perturbations are challenging to decipher within the context of the whole organism. Advancement in imaging and multiomic approaches are beginning to address these challenges. The explosion in the development of informatic tools is beginning to allow understanding of functional cross talk between members of complex ecosystems. Advancement in fluorescence-enabled imaging technologies such as combinatorial labeling and spectral imaging-fluorescent *in situ* hybridization (CLASI-FISH) allows us to visualize spatial structures of holobiont members and hypothesize their functional roles (1). Metagenome-assembled genomes (MAGs) allow cataloguing of metabolic capacities of individual microorganisms present in the holobiont and can be used to infer the key roles of individual members and logistics of spatial structuring (2). The transcriptome sequencing (RNA-Seq)-based metatranscriptomic approaches and metaproteomics methods allow a glimpse into a set of genes expressed and proteins encoded under a given set of conditions, respectively. Rapid turnover of RNA molecules and regulation of proteins via posttranslational modifications can compromise the correlation between transcriptomes and proteomes, making it difficult to decipher the response to change in phenotype (3). Moreover, novel biochemical transformations of genome-encoded metabolites by holobiont members result in new functions, but these modifications are difficult to predict using DNA sequencing-based methods. This is where metabolomics serves as a complementary tool and can fill the gap (4). Metabolomics includes analytical approaches to inventory and identify the collection of chemicals produced in a biological system. Since metabolites are essentially a functional readout of a biological phenotype, metabolites inform upon the functional state of a system as they serve as signals mediating chemical cross talk between members of the holobiont. But metabolomics also suffers from its own challenges. If we sequence the (meta)genome of an organism, we know the genes for which we can query the expression under a given set of conditions. But a similar luxury does not exist for metabolomics. In a typical metabolomics experiment, upwards of 5,000 metabolite features are detected, but only a small fraction can be annotated. Interrogation of which of these metabolites are produced by the host and which are produced by the associated microbiome is an arduous task. That we can neither predict nor annotate the complete set of metabolites produced by a member of a holobiont represents a major bottleneck to deciphering the chemical contribution of a given member of a holobiont to host health and microbiome fitness. Addressing this bottleneck is important to elucidate mechanisms of chemical cross talk responsible for structuring of the microbial community in a holobiont. Herein, the specific traits of a select community structure that allow it to resist dysbiosis, promoting holobiont health, and the specific traits of pathogens that allow them to colonize and result in dysbiosis will allow us to develop probiotic communities to modulate holobiont health. These are the fundamental mechanisms that my laboratory aims to uncover in environmental holobionts such as corals, as well as the opportunistic bacterial pathogens in human infectious diseases, through comparative metabolomics and collaborative multiomic approaches. The relevant questions and approaches to uncover metabolomic signatures of microbial processes from healthy and diseased coral holobionts are discussed below.

## OMICS APPROACHES IN HOLOBIONT SCIENCE

The coral holobiont represents a multipartite relationship between the coral host, the endosymbiotic dinoflagellate algae termed Symbiodiniaceae, the coral microbiome, and the dynamic coral reef environment (5, 6). Coral surfaces are lined with mucus, a resource-rich habitat that serves as a host for a milieu of bacteria, archaea, viruses, protozoa, and fungi. In the coral holobiont, the intimate symbiosis between the coral host and Symbiodiniaceae, shapes the mucus and the microbial composition (6, 7). While the host provides a habitat and nutrients such as phosphorus and nitrogen, the Symbiodiniaceae provide oxygen and the majority of the carbon and energy flux to the host through photosynthesis. The microbes in the mucus influence host health as commensals providing a defensive barrier and also as pathogens overcoming this barrier under opportunistic conditions (8, 9). When one or more partners in this holobiont are exposed to an environmental stressor, events such as bleaching (ejection of endosymbiont) and diseases imperil the holobiont, often resulting in tissue loss. Cataloguing of worldwide spread in coral diseases in the last few decades has revealed resilience of select coral species and the role of endosymbionts and microbiome against environmental stressors (10). However, we do not understand the biochemical basis of this resilience and how it changes with the incorporation of opportunistic pathogenic species during disease. Since coral reefs worldwide are imperiled by numerous devastating diseases resulting from pollutants and climate disequilibrium, it is essential to apply omics strategies to tease apart “who” is contributing to the change and “what” the change is. Such knowledge will provide the foundation to build effective steps to first protect, and then to restore coral reefs in the face of rapidly changing climate.

**FIG 1:**
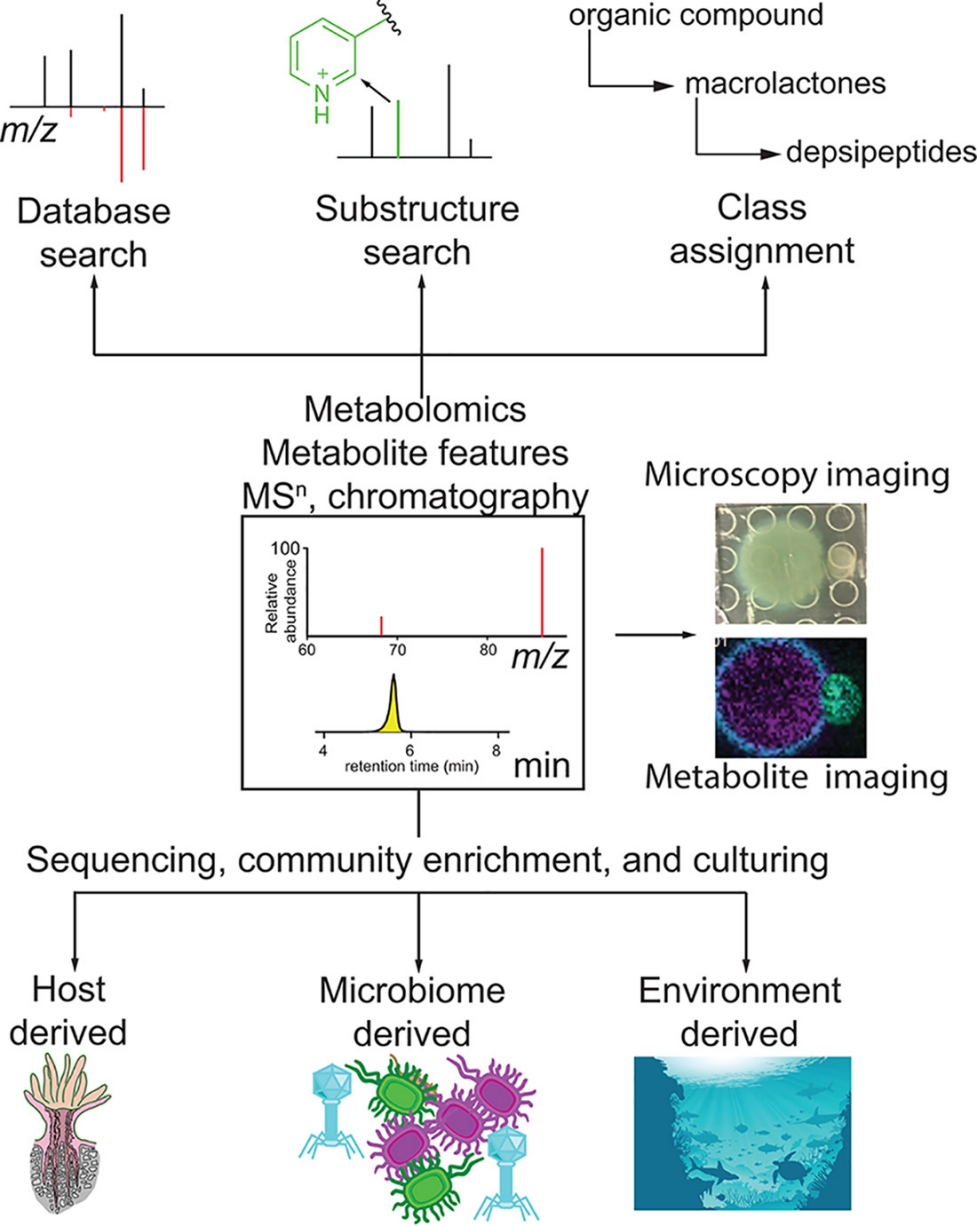
Omics and culture-based strategies to disentangle functional contributions of individual holobiont members. Sketch of coral polyp by Emerson L. Barrett (© Barrett, printed with permission).

Disentangling the roles of the microbiome, the endosymbiont, and the coral host itself in resilience and tolerance to disease requires a multifaceted approach. Inventory of microbial community composition can reveal shifts in microbiome architectures that serve as signatures of resilience or susceptibility. Similarly, we can identify microbial community signatures that are representative of disease (11). The mechanistic understanding of how these microbial signatures result in underlying phenotype requires elucidation of molecular markers of these phenotypes. Two approaches, when used concomitantly present potential in this respect. First, a top-down approach involves creating an inventory of all metabolite or microbes detected across different hosts. Whether the microbiomes and metabolomes of these hosts are correlated can be queried with methods such as Procrustes analysis (12). Then, by comparing the metabolomes and microbiomes, correlation networks (13) and neural network-based strategies such as mmvec (14) can link metabolites to specific microbes. Such data-driven approaches serve as hypothesis generation tools that require validation using model systems. Furthermore, the mechanistic understanding of the role of specific metabolites in dysbiosis or resilience to dysbiosis requires their isolation and structural and functional assignment. This process requires source tracking of metabolites and presents the biggest bottleneck in mechanistic studies linking molecules to a specific phenotype. However, the field is rapidly progressing with the development of *in silico* annotation strategies and metabolite source tracking (Fig. 1). We can now begin to assign the source of a metabolite peak to the producer in a multipartite organism through data-driven approaches and infrastructures to mine large omics data sets. These approaches are discussed below.

Spatial metabolomics, by correlating patterns of microbial community structure with metabolites, allows one to infer the biological source of metabolite and their phenotypic roles. Using this approach, Propionibacterium acnes was identified as the source of fatty acids via hydrolysis of triglycerides on human skin (15), microbiome-mediated bioconjugations of host-derived bile acids were discovered in mammalian gut (16), and localized differences in microbiome community within an explanted human lung were linked to production of microbial virulence factors (12). Direct mass spectrometry-based imaging approaches allow source tracking via colocalization of specific symbionts and detected metabolites (17). Next, with the explosion in *in silico* tools in the last 5 years, it is now possible to gain biochemical insights into the detected metabolome through substructure discovery (17, 18) and class assignment (19), enabling binning of the detected metabolome into biochemical pathways. The annotation of substructure allows one to predict biosynthetic pathways. The mining of these pathways in MAGs will provide a direct route to their biological source in the holobiont as we build the knowledge base of biosynthetic pathways.

A second bottom-up approach via comparative metabolomics of isolated holobiont members and the holobiont itself presents a traditional, but a direct approach. Herein, the individual holobiont members can be fractionated, enriched, or cultured. The endosymbionts have been fractionated from the coral holobiont mechanically with the use of a high-pressure jet stream and centrifugation. This process results in a relatively pure endosymbiont fraction with minor contamination from the microbiome. The metabolome of this fraction would allow source tracking of endosymbiont-produced metabolites in the coral holobiont and probe changes in these metabolites upon perturbations. Indeed, lipid biomarkers of thermotolerant endosymbionts have been proposed using this methodology (20). Finally, culture-based approaches will allow source tracking of microbial partners, and a rapid boom in microfluidics-based approaches now enables isolation of the unculturable bacteria (21). In this respect, a central repository of coral-isolated microbes has been proposed with the goal of development of coral probiotics, but currently no databases exist for coral-associated bacterial metabolomes (22). The marine environment is a significant source of the coral microbiome. Thus, metabolome repositories and infrastructures to coanalyze data sets acquired on isolated cultures from marine environments will be fruitful for metabolite source tracking in complex holobionts such as corals (23, 24). These approaches when combined with fluorescent probe-based visualization of *in situ* community structures (1) and mass spectrometry-based *in situ* imaging of metabolite gradients allow visualization of microbial chemotypes and source tracking, but remain to be developed for coral holobionts (17). We are excited to collaboratively employ such a data-driven top-down approach through profiling of microbiomes and metabolomes and a fractionation and culture-based bottom-up approach as the next important step to query the roles of coral microbiomes and their defense mechanisms in resilience, and susceptibility of corals to diseases.
